# Draft Genome Sequence of *Streptomyces* sp. Strain ICN903, Isolated from a Seaweed

**DOI:** 10.1128/mra.00635-22

**Published:** 2022-08-18

**Authors:** Albert Joshua Sam, Sangeetha Nallathambi, Samuel Gnana Prakash Vincent

**Affiliations:** a Centre for Marine Science and Technology (CMST), Manonmaniam Sundaranar University, Kanyakumari District, Tamil Nadu, India; University of Arizona

## Abstract

Here, we report the whole-genome sequence of the actinomycete *Streptomyces* sp. strain ICN903, which was isolated from seaweed of the genus *Botryocladia*. The whole-genome assembly contained 6,122,654 bp with 73% GC content. In total, 19 biosynthetic gene clusters (BGCs), including polyketides and terpenes, were predicted within the sequenced genome.

## ANNOUNCEMENT

Actinomycetes have been a limitless source of natural products especially antibiotics against infectious pathogens (1). Marine actinomycetes are the ongoing sources of novel bioactive compounds (2, 3). Tapping these microbes for new classes of compounds is inevitable until viable alternates are proven against raising microbial resistance (4).

The actinomycete *Streptomyces* sp. strain ICN903 was isolated from a *Botryocladia* seaweed collected from Pozhikarai beach, Tamilnadu, India (8.105144 N, 77.404993 E). The seaweed samples were washed with seawater to remove soil and surface residues. One gram of *Botryocladia* seaweed’s holdfast was aseptically separated, grounded, suspended in 2% NaCl to prepare serial dilutions and subsequently spread on Actinomycete isolation medium (AIM) agar plates (supplemented with filter sterilized 10 μg/mL nystatin and 50 μg/mL cycloheximide). *Streptomyces* sp. strain ICN903 was one of the numerous powdery actinomycete colonies that were found after 7 days of incubation at 30°C. To obtain axenic cultures, isolates were subcultured on fresh AIM agar plates with incubation at 30°C. The isolated colonies were kept in 20% glycerol at −20°C. For primary identification, 5-day-old axenic ICN903’s aerial mycelia were scraped from the AIM agar plates and the genomic DNA was isolated using the NucleoSpin Tissue kit (Macherey-Nagel) following the manufacturer’s instructions. The 16S rRNA was amplified with 2 μL template DNA within 20 μL of PCR solution containing 12 μL of *Taq* Master Mix (2× *Taq* buffer, 1.25 U *Taq* DNA polymerase, 0.4 mM dNTPs, 3.2 mM MgCl_2_), 0.25 μM forward primer (63F), 0.25 μM reverse primer (1387R) under the reaction conditions of 3 min initial denaturation at 94°C, 30 cycles of 94°C for 30 s, 60°C for 30 s, 72°C for 1 min and a final extension at 72°C for 10 min. The 16S rRNA gene (GenBank accession number ON738581.1) of this actinomycete was found to be closely related to the Streptomyces fenghuangensis strain GIMN4.003 (GenBank accession number GU356598) with a similarity of 95.38% by comparing with the EzBioCloud database (5).

The genomic DNA was extracted from the biomass of 7-day-old AIM agar plate culture using the HiPurA Streptomyces DNA purification kit (HiMedia Laboratories, India) following the manufacturer’s recommended protocol. Sequence libraries were created using the Nextera XT DNA Library Preparation kit (Illumina). The whole-genome sequencing was carried out using Illumina HiSeq 4000 system (paired-end 150 bp). A total of 18,821,928 bp reads were obtained within 2,842,111,128 total read bases. Adapter trimming was carried out with trimmomatic (v 0.36) (6), and quality was checked using FastQC (v0.11.5) (7). The read assembly was carried out using SPAdes v3.15.0 (8). Default parameters were used for all the tools, except where otherwise noted. After assembly, the genome consisted of 6,122,654 bp in 596 scaffolds. The scaffold N_50_ was 16,807 bp. The GC content was 73%. The completeness of the assembly was checked with BUSCO (v3.0.2) (9). There were 121 (97.58%) complete and single-copy BUSCOs, 2 (1.61%) complete and duplicated BUSCOs, 1 (0.81%) fragmented BUSCO, and no missing BUSCOs. The annotation of the genome sequence with NCBI Prokaryotic Genome Annotation Pipeline (PGAP) v6.0 (10) identified 5,809 total genes (CDS), 5,615 protein-coding genes, 3 rRNA genes, 65 tRNAs, 3 ncRNAs, and 123 pseudogenes.

Different classes of biosynthetic gene clusters (BGC) were identified using the online tool antiSMASH v 6.0 (11). Within 28 regions, 19 known BGCs were identified, which included ribosomally synthesized and posttranslationally modified peptides (RiPP), terpenes, non-ribosomal peptide synthetase (NRPS), and polyketides. Clusters with 100% similarity, including lagmysin, pristine, geosmin, ectoine, keywimysin, and alkyl-resorcinol were detected within the *Streptomyces* sp. strain ICN903 genome sequence. Deeper analysis and mining of the encoded gene clusters are required to find novel pharmaceutically important secondary metabolites.

### Data availability.

The draft genome of *Streptomyces* sp. strain ICN903 has been deposited in GenBank under the BioProject accession number PRJNA802313, the BioSample accession number SAMN25525657, and the GenBank accession number JAKMAY000000000. Raw sequencing data were deposited in the SRA with the accession number SRR17901780.
